# Provider, father, and bro – Sedentary Māori men and their thoughts on physical activity

**DOI:** 10.1186/s12939-016-0313-0

**Published:** 2016-02-04

**Authors:** Isaac Warbrick, Denise Wilson, Amohia Boulton

**Affiliations:** Taupua Waiora Centre for Māori Health Research, School of Public Health & Psychosocial Studies, Auckland University of Technology, Private Bag 92 006, Auckland, 1142 New Zealand; Whakauae Research Services Ltd, PO Box 102, Whanganui, 4540 New Zealand

**Keywords:** Māori, Mens health, Minority health, Physical activity, Exercise, Barriers, Health promotion, Focus groups

## Abstract

**Background:**

Māori (indigenous peoples of New Zealand) men have a disproportionate prevalence of lifestyle-related illnesses and are targeted for national physical activity initiatives. While physical activity impacts on physical and mental health and overall wellbeing, current approaches to health promotion often lack cultural relevance. Having better understanding and incorporating relevant cultural values and motivators into program designs could improve the success of health initiatives for indigenous and minority men. Nevertheless, little is known about Māori men’s preferences, attitudes, or perspectives about physical activity, which are often interpreted through a colonized or dominant Western lens. Understanding perspectives of those groups whose values do not align with dominant cultural approaches will better equip health promoters and trainers to develop relevant community initiatives and private programs for indigenous and minority men.

**Methods:**

An indigenous research approach informed a qualitative study with 18 sedentary, ‘overweight’ Māori men aged 28 to 72 years. From 2014 to 2015 these men participated in three focus group discussions aimed at understanding their views about physical activity and exercise. Data were thematically analysed and interpeted using a Māori worldview.

**Results:**

Four key themes were identified – Cameraderie and ‘Bro-ship’; Adulthood Distractions and Priorities; Problems with Contemporary Gym Culture; and Provider Orientation. Key motivators for physical activity included a sense of ‘brotherhood’ in sport and physical activity and accountability to others. Participants reported the need to highlight the value of people and relationships, and having an orientation to the collective to enhance physical activity experiences for Māori men in general. Modern lifestyle distractions (such as being time deficient, and family responsibilities) along with other priorities contributed to difficulties incorporating physical activity into their daily lives. In addition, particular aspects and characteristics of the modern fitness culture and gym environment acted as barriers to adherence to physical activity.

**Conclusions:**

Sedentary Māori men understand the importance of physical activity well, and have a desire to be more active. Nevertheless, they find it difficult to do so while balancing other priorities, especially cultural obligations to community and whānau (immediate and wider family). This research provides valuable insight for those promoting physical activity or designing health initiatives so that they better resonate with indigenous and minority men.

## Background

*Tama tū, tama ora. Tama noho, tama mate - He who is active lives. He who stays still dies*– Traditional Māori proverb

Regular physical activity improves physical health and overall well-being while also reducing the risk and effects of physical and mental illness [1–4]. Nevertheless, reduced physical activity is a factor strongly associated with a growing prevalence of cardiovascular disease, diabetes, and even mental illness, and continues to become more widespread as modern lifestyles become less dependent on physical activity [5]. Despite global public health efforts to improve physical activity levels, sedentary behaviour in developed and developing countries continues to be a concern [6]. While it is common practice to blame a lack of individual willpower or laziness as the cause for such behaviours [7], the reasons are more complex and require examination of social, cultural, and environmental factors that create or contribute to barriers to being physically active [8, 9].

As with other indigenous groups around the world, and indeed most ‘non-Caucasian’ ethnicities, Māori (the Indigenous peoples of New Zealand) are disproportionately affected by lifestyle-related illnesses associated with sedentary behaviour [10], and mortality as a result [11]. Such disparaties between Māori and New Zealand Europeans (NZEO) in preventable illnesses are strongly associated with broader socio-cultural/political determinants of health and illness such as income, housing, and education. These are experienced by many indigenous peoples, including displacement from ancestral lands, traditional food sources, and wider family networks, poverty, discrimination, and unemployment [12, 13]. These broader determinants, underlie the behaviors we deem as causative to chronic illness at an individual level, such as higher alcohol consumption, high sugar intake, increased stress, and physical inactivity [14–16]. These wider determinants of health are recognized in the ‘Whānau Ora’ (family wellbeing) strategy in New Zealand, a government-supported strategy that employs a top down approach aimed at working accross sectors to deliver integrated services to improve these determinants of health behaviours within families rather than the usual approach of focusing on the behaviours themselves. For that reason, recent physical activity initiatives and innovations in New Zealand have focused on increasing cultural and social relevence for Māori, utilizing principles of Whānau Ora and traditional Māori knowledge [17, 18].

Nonetheless, little is known about the thoughts and preferences of sedentary Māori men toward physical activity and exercise – often the only options available are initiatives like private fitness centres that are underpinned by dominant neoliberal ideals. Characterized by individual responsibility, and the belief that inequality is inevitable as a consequence of choice [19], neoliberal ideology is so embedded in modern health policy and practice that it can be difficult to comprehend, let alone accept, an alternative ideology [20, 21].

A large body of literature has been dedicated to understanding thoughts about, motivators for, determinants of, and barriers preventing physical activity for the population at large. Other studies have focused on a variety of demographics including the elderly [22], high school students [23], and ‘minority’ [24] or immigrant [25, 26] groups. However, there are very few studies specifically on indigenous peoples, particularly indigenous men’s perspectives about physical activity and exercise. Although studies on the barriers and facilitators for physical activity often identify universal themes common among all people, such as competing priorities and lack of motivation [27–29], consideration needs to be given to circumstances unique to gender, ethnicity, religion, and age. Furthermore, once people have had an opportunity to share their views, it is important that these are interpreted in a way that reflects the cultural beliefs and values of those who gave them. While this can be difficult, understanding the unique perspectives of a population targeted for health interventions is essential to maximize acceptance and adherence to a lifestyle change.

The views of indigenous peoples are of particular interest to our research group as indigenous ‘voices’, values, and worldviews have often been silenced or laid aside at the expense of prevailing dominant discourses, although many are now challenging these practices [30–33]. Prior to colonization, Māori and many other indigenous peoples were physically active in order to sustain life; families worked, migrated, fought, and performed in tribal groups as survival was seen as a collective pursuit. Yet, current discourses stress ‘personal responsibility’ with regard to health and physical activity [20], placing blame on indigenous peoples for the marked inequities in the health status and health outcomes they suffer. Frequently, Māori are subjected to negative stereotypes and deficit explanations that direct fault at Māori for being fat, unhealthy, and sedentary along with accusations that they make poor lifestyle choices [34]. Such attitudes disregard the pervasive influences of colonization, contemporary socio-cultural and environmental factors, and genetic factors that may contribute to physical inactivity. Like most researchers in this field, we agree that individuals have a responsibility to be physically active, but current finger-pointing approaches and imposing ‘personal responsibility’ neglects the complexity inherent in this issue and the various barriers that influence one’s choice. Moreover, it does little to motivate healthy behaviours or lead to lifestyle change on a significant scale [35].

Through focus group discussions we aimed to understand Māori men’s past and current patterns of physical activity, why they had become sedentary, and what changes they wanted to make to transition to and maintain a physically active lifestyle. As the participants were about to commence a formal 12 week exercise program as part of a wider study soon after the focus group, we also sought to understand their expectations for this exercise intervention prior to its implementation.

## Methods

The current research took place in the context of a larger mixed-methods study spanning 2014 and 15, *The Best Exercise for Māori Men,* which examined the impact of participating in a structured 12-week ‘culturally-enhanced’ exercise program on (a) physical markers of health and illness (metabolic blood markers, aerobic fitness, and body composition), and (b) subjective well-being. For the purpose of this study, culture was the values, beliefs and practices informed by an indigenous worldview that guide people belonging to a common group in their everyday lives. Therefore, the program was informed by the values associated with a holistic worldview (whereby family and caring for others are important, for example), the importance of relationships were privileged, and incorporation of a collective approach to the exercise program rather than focusing on a group of individuals. Ethical approval for this study was obtained from Massey University’s Southern A Ethics Committee (Application 12/19). All participants provided written informed consent to participate, have discussions recorded and transcribed, and for results to be disseminated and published.

We aimed to recruit between 15 and 20 Māori men using network sampling from family, work, or church networks and came from the Horowhenua and Manawatū Provinces in the lower part of New Zealand’s North Island. It was anticipated that this number would achieve data saturation. Flyers advertising the study were placed in a university’s Māori department, and at various other locations (such as workplaces or health services) where relatively high numbers of Māori frequented. The inclusion criteria for the focus group discussions were Māori men who self-identified their ethnicity as Māori, had a BMI over 25, and were ‘sedentary’, participating in a 30 min block of pysical activity or exercise less than twice a week, including employment which was ‘physically demanding’. Other than 4 participants who responded to the flyer alone, the cohort of men ho participated was mostly made up of small peer groups from different networks; 2 or 3 men who either worked together, played social sport together, went to church together, or saw the flyer at University and invited a friend to respond with them. Most of the men worked in educational, managerial, or administrative-type employment and none of the men who participated in the discussions had (self-defined) ‘physically demanding’ employment. Those who participated were from a variety of communities, workplaces, education levels, and lifestyles.

We undertook three focus group discussions with ‘sedentary’ Māori men; two on marae (cultural meeting places), and one at an indigenous learning institution. Participants were welcomed with a whakatau (formal Māori welcome ceremony) at each venue. Following Māori cultural protocols for hospitality toward visitors and the cultural significance of food as part of welcoming people, participants were then invited to have a meal with the researcher. The marae, a traditional Māori gathering place with its ancestral meeting house (wharenui), was an ideal setting to begin the study and introduce participants to researchers and to each other. This initial gathering set the tone for the kaupapa (values and principles) Māori cultural approach that were used in this study. The key cultural principles of wanaungatanga (relationships and connections) and manaakitanga (generosity and support) underpinned this study, and guided recruitment and interaction with participants, facilitating focus group discussions, and the data management and its analysis and interpretation.

Whānaungatanga highlights the value of kinship, relationships, and shared experiences, placing value on responsibility and accountability to the collective group. In this case, it meant establishing familial links and treating participants as whānau (extended family). Through the process of introductions during the formal welcome, researchers and participants recognized mutual family and cultural ties, which enhanced participants’ sense of accountability to each other.

Manaakitanga relates to reciprocation that runs deeper than simply giving and receiving. It includes a deep sense of hospitality and the importance of enhancing the mana (prestige and status) of others. In the case of this research, participants were seen as equal (or even greater) contributers to the outcomes of the research, and the knowledge they shared was treated as taonga (a treasure). This meant that research and career agendas of the researchers, though important, came second to the aspirations of participants and their whānau. Utilization of kaupapa Māori research approaches have been discussed extensively elsewhere [36, 37].

The focus groups lasting 55 min on average were designed to have a relaxed atmosphere to enable participants to freely express themselves and encourage open discussion. The lead researcher, a Māori male, faciliated the discussions and established relationships with participants by promoting a culturally appropriate environment for discussion following principles of manaakitanga and whanaungatanga. The semi-structured questions began with a key question, while enabling more specific questions to explore areas arising from the discussion. These included the following:

- What are your thoughts about physical activity?
- What kind of physical activity have you done in the past?
- Why are you less physically active than you were in the past?
- What factors stop you from being physically active or engaging in exercise?
- What types of exercise/physical activities do you prefer? Which do you avoid?
- Do you think that being Māori influences your decisions to exercise at all? If so, in what way?
- What factors would motivate you to exercise more (be more physically active)

Audio recordings of the focus group discussions were transcribed verbatim, and the transcriptions were checked for accuracy and thematically analyzed [38]. An inductive approach was used for coding which was completed by the first and second authors separately, who then met together to discuss and reach a consensus regarding final coding and themesOnce codes were identified, these were sorted and organized into themes and concepts emerging from the data. That is, codes were compared and contrasted and similar codes classified together to produce four higher order themes and sub-themes which will be outlined later [38]. The third author then reviewed the data and the themes generated during this process to confirm inter-rater reliability. Themes were confirmed when data saturation occurred – that is, there was no more new information. Particular attention was paid to understanding cultural aspects of sedentary behaviour.

## Results

Eighteen men aged 28 to 72 years old (mean age of 38.7 years) from relatively diverse backgrounds participated in this phase of the study and, while from a variety of Iwi (Māori tribes), they were at the time living in Ōtaki or Palmerston North in the southern part of the North Island of New Zealand. Participants were recruited over 6 months. All men were classed as ‘sedentary’, and were considered with a BMI >25, to be ‘overweight’ by standard BMI cut offs. However, a few men regularly participated (not more than once a week) in social games of sport (rugby, touch rugby, or basketball), though none were involved in active transport (walking, running, or cycling to work for example) or had scheduled exercise times during the week. Four key themes emerged from the data: *Cameraderie* and *‘Broship’*, *Adulthood priorities and distractions*, *Problems with contemporary gym culture, and Provider Orientation.*

### THEME 1 – Camaraderie and ‘Broship’

Camaraderie or ‘broship’ had an important role in motivating participants to be physically active. They talked about how much ‘easier’ it was to exercise with a friend or a ‘bro’.

*When we’ve got the bros motivating us, sweet as, easy.*

Participants often referred to the ‘bros’ that were around them, whether it related to physical activity or not, along with the physicality and interactions they had with male peers. Evident was an unwritten but strong accountability to the ‘bros’. This was apparent in one participant’s recollection about his time in the army:

*It was a strenuous time of my life. I guess I was fit, but more than that it was the fullas [fellows]. I spent 5 years in there* [the Army] *with some awesome fullas. … That’s what I miss – that’s deeply connected to my physicality – is to be with fullas, coz* [because] *they just hold you accountable without saying a word.*

Thus, motivation to exercise was largely intertwined with their ability to interact with peers while being active. Having a social group helped to get “*over the hump*” when motivation or energy was low. A lack of peer support contributed to a loss of motivation during previous attempts to attend the gym.

*…when you feed off each other, that’s what whānau* [immediate and extended family] *is. It’s to feed off each other. That’s how I’m gonna get past the hump. If I can do that, that’s better than going over the hump alone, to get there* [goal] *with each other.*

#### Accountability and obligation to others

Participants had a strong collective orientation, whether it was as part of a military unit, sports team, or group of friends. Apparent in this orientation was a sense of accountability and responsibility to the group and its members to persevere and do their best.

*When I know I’ve got a role and a responsibility and there’s a goal at the end, I can’t help but think, “Man I’ve gotta do some business.” And so I know if the brothers are depending on me, I’ll go out of my way to ensure that I’m not the guy that drops the ball.*

This sense of accountability to others in a group superseded individual aspirations, and its absence made motivation to exercise and continue it difficult. Not performing their best negatively affected the entire group and it was important to avoid this. This sense of accountability to the ‘bros’ was a noticeable trait among Māori.

*…it’s not just accountability, but what comes with that is aroha* [unconditional regard or love]*…Māori soldiers led the way in that kaupapa* [purpose], *especially in the infantry, because I think they have that natural thing to fight for each other.*

Accountability to the group, and its importance as a motivating factor extended to the men’s roles in their whānau and communities. Past physical activity experiences included working on a task as part of a group, family, or community, including sport (usually team sports). Mostly, it was physically demanding work, often not considered ‘exercise’ in the modern sense, such as gathering or preparing food, chopping wood, or performing work around a relative’s farm.

*For me it was community as well. As part of church we’d go diving as a community, chop wood as a community, so we did things that we never thought were fitness…community was important.*

When talking about earlier life experiences, participants’ memories of physical activities were almost always linked to community or group involvement.

#### Challenge and ‘constructive’ competition

Competition arising from interactions with other men was a main culturally relevant ‘driver’ for doing physical activity. However, this competitiveness was directed towards enhancing their own performance and others in the group, and generally not directed at winning over others.

*Although we’re not competing against each other, men, not just Māori men, but men I reckon have a competitive streak in us. We want to push ourselves and we look at the guy next to us, and if he’s doing a little bit more, we want to do a little bit more too. It’s just the way we are, we’re built like that.*

This competition was often expressed as a chase – such as chasing an older family member who was better at sport than they were when they were young, or trying to keep up with younger players when they attempted to play sport in adulthood.

### THEME 2 – Adulthood priorities and distractions

Prior to adulthood, participation in sport was extremely important to these men, often taking precedence over education and other activities. Once they got older, work and family commitments became a priority, leaving little time or energy for sport or other physical pursuits.

*When I was a young fulla, I didn’t know anyone who didn’t play sport. All my mates played sport and we played at least three sports … but now I can talk to most of my mates and half don’t even play sport. They’re happy to sit at home.*

During childhood, sport was a focus so being physically active was a default and being sedentary was abnormal. Participants also felt that childhood physical activity was required for entertainment, transport, and even contributing to daily living (such as gathering food).

*That was mahi* [work]*, it was not exercise. It was mahi, but you were being exercised.*

*We did things we never thought were fitness.*

Physical activity was not only a normal part of everyday life during childhood but it seemed as though no effort was required to be active as it occurred naturally. This doesn’t seem to be the case in adulthood however, where, despite numerous attempts to be involved in some sort of regular physical activity, whether at the gym or by another means, various distractions in their life got in the way, causing them to lose focus.

*Something would happen, like in the family, and I’d lose focus. Normally it was injury that* [had] *done it for me or a relationship problem that sort of distracted my focus. It’s hard to get back on that track, and the mind-set it’s hard to keep it going consistently.*

Older men particularly reported that old sports injuries (such as knee injuries) limited their physical activity.

#### Whānau a distraction and priority

Participants’ commitment to their children highlighted the concern they had for their family’s needs and the priority they placed on their roles as fathers and partners. Fulfilling this role successfully required a reduction in their physical activity and redirecting resources to the family, ahead of themselves.

*…especially now that I have children and family. I think of what they’re gonna be missing out on. Should I go away for an hour doing some exercise..?”*

A healthy relationship with their partner was of high importance because this positively impacted the men’s motivation to be physically active, while relationship issues such as break ups could have negative impacts.

*If you look at success, you’d see that the majority of successes with Māori men occur when the relationship is strong and the whānau becomes stronger. When the relationship between the two is strong it doesn’t matter if it’s the male’s strength or the woman’s strength, but where that relationship is able to build and grow then the whole whānau and the whole iwi becomes strong.*

Closely tied to family-related distractions were financial barriers. The men reported that being active cost a significant amount of money. Gym and other costs were seen as depriving the family which made it particularly hard for men to justify the costs of formal exercise programs.

*I can’t afford $80 a month for some of the gyms, let alone pay someone to help me work my way around the gym. I’ve never thought about putting that much money into the gym.*

Family and work responsibilities also contributed to participants being time deficient.

*For me my family is my priority and all my four kids play sport…and they all play on different days…so I’ve had no physical activity for 9 months…I look back now and just thinking about what everyone has been saying made me think that I really went downhill about 4 years ago when I stepped up into a new job and got busy, and my kids got really active in sport and my wife started working as well. It’s just a matter of trying to fit physical activity in your day…there are 24 h in the day, I need 28.*

#### Technology a distraction

References to the physical nature of their upbringing in contrast to their current more sedentary lifestyles was imbued with concern about the overuse of technology, particularly among their own children. Participants noticed a shift from outdoor, physical recreation to mostly indoor, sedentary activities over their lifetime, and particularly noted the impact increasing use of technology had.

*It’s just so much different from what we we’re doing…when we were young we’d just go possum hunting; eeling; go down to the river* [and] *cook spuds in the fire,* [or] *go fresh water crayfishing up in the hills, run everywhere. Now they’ve got machines and buttons and that seems to be the recreational pathway at the moment. But when we were young we had to make our own fun …I try and tell my daughters but they’re just used to being on the PlayStation”*

### THEME 3 – Problems with Contemporary Gym Culture

There were a number of factors that were seen as problems with going to a gym. While some men enjoyed the gym in the past, others found it ‘boring’ and considered it a last resort, preferring instead physical activities that usually involved sport. A key deterrent to using the gym for some men involved feeling like being in a *“fish bowl”*, which invariably affected performance because of their ‘public’ nature.

*There’s all these mirrors and you’re looking at yourself, and you’re worried that others are looking at you.*

*I perform better at a place where I’m not getting looked at and can let it all hang out.*

The types of people working in gyms compounded these feelings, particularly as they perceived they had little in common with these Māori men.

*You walk into a gym…and a five foot tall girl that does ballet is the personal trainer you can talk to. Really?! Nah* [no]*. It’s not gonna happen bro!”*

#### Needing the ‘right’ teacher

Despite the availability of exercise-related information on the internet, participants indicated they lacked the education and ‘know-how’ required to exercise effectively. This was compounded by conflicting health promotion messages, making it difficult for them to navigate the opposing information and messages.

*I jumped online to learn how to run properly and there’s all these different random techniques. So it’s hard to know what’s going to work.*

Those who had used a personal trainer or fitness instructor found their expectations of the trainer were rarely met. However, others provided examples of the characteristics of helpful, informative, and motivating coaches, teachers, and instructors.

*I had an awesome coach who showed me all these different techniques, he learned about my strength, my weaknesses, he really got to know how I played and developed me into something better, and it was awesome.*

Participants wanted to gain knowledge from those who trained or instructed them, which they wanted to pass on that knowledge onto friends and whānau.

#### Falling off the wagon

Participants reported the costs of gyms was a ‘rip off’, with many stating this money would be better spent on their whānau. While most had attended a gym or participated in an exercise program, they reported rarely receiving ongoing support, making it difficult for them to connect exercise at a gym to a worthwhile purpose, outcomes, or rewards. Also missing was a sense of competition and challenge in contemporary gym settings.

*There’s no actual competition. It’s not like you’re competing, you’re not chasing. If there was something to chase; if there was a win at the end. But the gym just seems like an ongoing thing that never ends.*

### THEME 4 – Provider Orientation

The main study’s intervention was an opportunity for participants to make regular physical activity a lifestyle and lasting habit, although participants expressed concerns about being able to maintain habits and continue to progress when it came to an end.

*That’s what I’m kind of afraid of after these 12 weeks. Am I going to go back to my* [previous] *lifestyle, or am I going to find something else to do?*

Nevertheless, the invitation to participate in the intervention reminded participants how being active improved their overall wellbeing, although their motivation was generally connected to their everyday life and workplace activities, along with their desire to live longer for their children and whānau.

#### Mahi (work) as physical activity

Men frequently associated physical activity with being a provider – whether for their own immediate families or for the wider community and extended families. Participants connected physical activity to their work and their everyday life. Being able to provide for others was valued, more than apparent physical fitness for themselves as individuals.

*The people I loved and adored in my life looked exactly like that* [pointing at one of the ‘bigger’ guys in the room]*…They’re the people I put on a pedestal because they were the people I saw at the hangi* [underground ‘oven’ for cooking] *pit, they were the people gathering the pauas* [shellfish]*…Bro, our best divers in our whānau didn’t look like Sonny Bill Williams* [well known professional rugby player], *but they fed their entire hapū* [sub-tribe] *with their mahi.*

#### Living longer for whānau

The important role Māori men have as fathers, and their desire to be healthy ‘role models’ for their children were key reasons to become more active. In some cases, children motivated lifestyle changes such as quitting smoking. Many participants expressed wanting to live longer for their family, and recognised the impacts poor health would have on their whānau.

*When the man loses his health and loses his way and dies early then the whole whānau falls down.*

#### Overall well-being

Getting fitter, losing weight, gaining confidence, and feeling better about themselves were the men’s expectations and hopes when they agreed to participate in the intervention part of the study that would follow these discussions, but most had specific functional goals.

*Bro there are things that just aren’t happening right. I’m getting tired carrying my fishing net out into the river. I can’t hold my breath as long underwater when I’m doing a dive. I can’t shop online because they don’t have my size. There’s little things that just remind me.*

Participants reported that being physically inactive spread into other parts of their home and work lives, while ‘being fit’ and active improved their psychological and spiritual well-being.

*If we’re not exercising physically I know I sit on my couch and bark at my girls rather than getting up and parenting like I should…When I was physically in shape I didn’t have a problem*.

*If you’re fit you’re more mentally alert. Your spirit and everything. Being physically fit has a lot to play in our lives as Māori. The more fitter we are, the better Māori will be.*

## Discussion

*He aha te mea nui o te ao? He tangata, he tangata, he tangata – What is the most important thing in this world? It is people, it is people, it is people*– Traditional Māori proverb

Despite Māori men being a main target of public health interventions in New Zealand, this study appears to be the first to identify facilitators and barriers to physical activity within a cohort of Māori men. The results from our study with a group of sedentary “overweight” Māori men indicates that there are a number of factors to consider when developing health initatives for indigenous peoples, and for other ethnic minority groups globally. The cameraderie with other men, the various distractions and priorities in their daily lives, the disconnect with contemporary gym and fitness culture, and the tension between being a provider and being physically active are crucial considerations for planning meaningful physical activity and reducing inequities in health for this group of men. Although many aspects of the themes identified are not unique to Māori, indigenous, or minority groups, we feel that our findings and the findings of others suggests that a Māori worldview and way of ‘being’ is crucial to developing approaches that are ‘mana enhancing’ and strengths-based. Such approaches pay particular attention to the strengths of men rather than the negative stereotypes which they are too often defined by [39].

Whakapapa (genealogy and history) is an important aspect of Māori culture that links past, present, and future. All the men in our study talked fondly about being very physically active before adulthood. The men recounted participation and excellence in sport, particularly team sports, in their younger years that provided ‘challenges’, motivation, and drive while also meeting a social and competitive ‘need’. However, many also recalled the difficulties of being expected or ‘forced’ by parents and other family members to do chores (such as helping on a relative’s farm, or gathering sea food). Nevertheless, these seemingly negative experiences were told with gratitude and laughter rather than resentment. The men found these commonalities quite humorous. Fittingly, studies about intercultural communication in New Zealand have also observed that Māori men are far more likely to look back on past times and share, with a distinctive humor, experiences they had with others, in contrast with Pākehā (New Zealand European in this case) men who seem to recount experiences at an indiviual level [40]. Other qualitative studies have also observed, with minority male groups, this fond reminiscence of past participation in sport and exercise [24]. Ultimately, these recollections highlight the contrast between unintentional physical activity in childhood, and the deliberate and significant effort required to regain and maintain physical activity levels in adulthood.

The cultural concept of whānau (extended family that is inclusive in nature, and may be comprised of either genealogical or members with a common purpose) is evident in the theme Cameraderie and Broship, whereby the men found comfort in the togetherness of the group. In addition, the collective orientation of Māori was evident in the men’s sense of obligation and accountability to the other men in their group. Furthermore, many of the men highlighted their responsibilities as father, husband, and provider as a main reason for not being physically active. In a neoliberal societal context where there is great emphasis on, and indeed an expectation of, personal responsibility for one’s own health and well-being, being busy as a father and provider maybe regarded as simply an excuse for not being healthy. While these expressions of concern and priority toward their family appears to be at the expense of personal health, it is also a commendable attribute and strength that reflects a high level of commitment to family and relationships, something that is valued greatly in Māori culture.

Men, and particularly indigenous and minority men, are often portrayed negatively in the media and by other institutions, particularly regarding parenting ability and interactions with children [41, 42]. However, like others [41], we found our Māori participants possessed a strong sense of responsibility, obligation, and sacrifice. Early European settlers’ accounts in New Zealand also document Māori men as being noticeably active and affectionate fathers [43]. Sacrificing personal well-being for family was also expressed by Māori men in other situations. For example, resisting urges to mourn during traumatic times so they can be a strength to family [44]. Others found that African American men also placed the fulfillment of their role as provider, father, and teacher of children at the top of their priorities, often at the expense of their own health [45]. This is not to say that the desire to fulfil the provider role is detrimental to one’s health, as the drive to be a successful provider for family has also been identified as a motivator for healthy behaviours [46]. Rather, this finding highlights the need for health promoters to consider, when developing initiatives for men, the depth of responsibility some groups of men have to provide and make sacrifices for whānau. Furthermore, consideration should be given to the important role that family has in the health of their men, and that spousal reassurance about time for exercise and physical activity is positive and encouraged as an investment in their whānau (family) health.

Māori men’s strong sense of responsibility and accountability to others extends beyond immediate and extended family to include the ‘bros’. ‘Bros’, a term from ‘Māori English’ that is commonly used by Māori to address friends and peers, is thought to reflect the importance of kinship and interconnectedness for Māori [47]. Accordingly, in all groups interviewed, ‘bro’ appeared to be the most preferred term when referring to friends, a group of friends or teammates (the plural ‘bros’), or when interacting with other members of the focus group. Using such a term serves to engender solidarity between speaker and hearer [48] while others suggest that it functions as a marker of ethnicity for Māori [49]. For this reason, the term ‘broship’ was adopted within our research group to explain the common theme which represented a uniquely Māori perspective of camaraderie, friendship, and brotherhood.

A sense of accountability to the bros was usually manifest when talking about experiences of encouragement toward peers and reciprocation of such encouragement, which in turn is closely tied to the principle of manaakitanga. Others have observed the ‘need’ for peer support accompanied by a sense of accountability to peers in African American men [24]. We also found that the enjoyment associated with physical activity was more closely linked to undertaking activity within a group of men than it was to any other factor, such as the type of activity, or where the activity was done. Similar notions have been proposed by Hammond and Mattis [50] on masculinity, where they found men constructed their identities and ideas about masculinity in relation to their responsibilities to family and others. In their study with Māori and Pakeha men, Hodgets and Rua [41] commented that “*mundane acts such as fixing cars, fishing or just hanging out…provide opportunities for maintaining relationships and cultivating a sense of trust, support and belonging* (p.165).” The Māori men in our study also indicated their identity as part of a collective, and their relationships were almost always tied to seemingly ‘mundane’ activities such as gathering food or labouring together for a common purpose. Once again, the effects of colonization and dominant cultural beliefs that promote a focus on ‘individual’ effort contribute to men feeling that the motivation and support they gain within a group of men means they then fail as a provider. Yet, whanaungatanga and kotahitanga (unity and collective solidarity) are guiding cultural principles that upheld a succesful social structure and saw Māori men achieve great feats in survival, provision, warfare, and navigation for many generations prior to colonization. Rather than a weakness, ‘broship’ was, and is an inherent aspect of success for Māori men. On the other hand, the isolation of Māori and indigenous people from their lands, culture, and tribal/extended family networks as a result of colonization, has contributed to the health inequities experienced by Indigenous peoples. Our study also indicates that current health promotion approaches that encourage attendance at a gym with strangers, with loud music through headphones, creates a disconnect from social interaction and support which further isolates these men. Thus, promoting physical activity based on messages of ‘personal’ responsibility will not likely resonate with Māori or be effective among indigenous and minority men.

In most cases, physical activity in adulthood was seen by the men in this study as a competing interest that would take time or resources away from more important priorities. Work was one such interest, but more than anything, personal pursuits such as physical activity were sacrificed to ensure children and family had adequate time and resources. In accordance with the ‘Health Belief Model’ [51], people are more likely to engage in physical activity if they feel that percieved benefits outweigh percieved barriers. We found that the reverse is also true; that people will be less likely to engage in physical activity if the percieved barriers outweigh the percieved benefits. Reducing the most recognizable barrier to physical activity (commitment to family) is obviously not the way forward. Instead, a more appropriate and acceptable strategy would be to focus on the benefits for family and deeper commitments to people. While the immediate reaction is to include whānau in an intervention, the value for the men of having the time with, and support from, the ‘bros’ suggests that whānau exercise groups may be slightly off the mark. Instead, our data suggests that while family must be considered and included in health promotion initiatives and wider aspects of an intervention, exercise groups for the men may be better attended if established as ‘men-only’. We also believe that employers and communities should consider supporting physical activity opportunities that include aspects of provision and community service. Nevertheless, further research is needed to establish an effective model for acceptable innovative interventions that meet the needs and priorities of indigenous men.

We started this paper with a whakataukī (traditional Māori proverb) that outlined the importance of being active. Another well-known whakataukī is particularly pertinent to the findings in our study: *He aha te mea nui o te ao? He tangata, he tangata, he tangata*. This whakatauki, translated as *‘What is the most important thing in this world? It is people, It is people, it is people’*, reminds us that above all things, people, their interactions and relationships are the most important thing to Māorir. Accordingly, all of the themes identified from our discussions reflect how Māori men are intrinsically linked to people and in some way or another explains why they were or were not physically active. Māori men’s collective orientation and sense of responsibility runs counter to the dominant discourse that focuses on individualism and individual responsibility. Continuing to approach physical activity from a stance focused on individual responsibility and attainment of individually focused goals inadvertently perpetuates the health outcome inequities that currently exist for Māori men. Persisting with physical activity initiatives grounded in individual responsibility is not equitable, and is unlikely to increase participation or reduce the burden of sedentary-related health conditions experienced by Māori. Planners of health promotion and physical activity initiatives would do well to consider how the messages they promote to indigenous and minority men, such as Māori, can be underpinned by a collective-orientation that considers the importance of family, relationships, and broship.

### Limitations

While this study has a relatively small sample of sedentary Māori men, it provides valuable insights into factors that enable and prevent Māori men from becoming more physically active. The men in this study were from a variety of backgrounds, lifestyles, workplaces, and communities within the Horowhenua and Manawatū provinces, yet were almost always familiar with the other participants personally, or with family members and friends. Although this degree of familiarity could be seen as a potential cause for bias, it is very common for Māori within a region the size of those where the interviews took place to be familiar with or related to (on some level or another) the other Māori individuals living within that region. Also, the men who participated in this study had accepted the invitation to participate in a 12-week exercise intervention, thus our findings may be limited to those who are already motivated and seeking to become physically active. It was also evident that those men who participated were all active in their childhood and youth, only becoming sedentary in adulthood. It is important to understand the perspectives of those indigenous and minority men who have never been physically active given the literature suggests ‘always-been-sedentary’ individuals have significantly different views about exercise than those who are active or even ‘recently-sedentary’ [29]. It would be interesting to see if ‘always-been-sedentary’ Māori men share similar perspectives to ‘recently sedentary’ Māori men in this study, or if they are more closely aligned to the views of ‘always-been’sedentary’ non-Māori men. Finally, while we interpreted the findings from an indigenous Māori worldview, it must be noted that there is diversity in views and values of those who identify as Māori.

### Implications

Although it is likely that the Māori men within this study would have more in common with non-Māori men than differences [52], the themes that are unique to a particular group are probably more valuable from a public health perspective. To elicit features unique to various groups, qualitative focus groups or interviews would be beneficial prior to developing interventions. Self-determination theory suggests that intrinsic motives (e.g. pleasure, enjoyment) rather than extrinsic motives (e.g. weight loss, appearance) lead to long-term participation in physical activity [53]. Therefore, developing interventions that give men an opportunity to interact with other men could improve the sustainability of physical activity and other lifestyle changes, regardless of what mode of physical activity is used.

## Conclusions

Four key themes were drawn from the views shared by sedentary Māori men prior to commencing a 12 week exercise intervention. The importance of people, relationships, friendships, and roles within family are intrinsic in these themes and link each theme together. This is an important aspect of a Māori world view, expressed in the proverb: *“He aha te mea nui o te ao? He tangata, he tangata, he tangata”*. Consideration of the collective orientation that is distinctively Māori and evident in these men’s accounts is important and may play a part in reducing the health inequities, associated with physical inactivity, experienced by these and other men.
